# Processing at-issue and non-at-issue content: Evoked and induced brain activities reveal early and long-lasting differences

**DOI:** 10.1371/journal.pone.0321953

**Published:** 2025-05-21

**Authors:** Anna Czypionka, Laura Reimer, Mariya Kharaman, Carsten Eulitz

**Affiliations:** 1 University of Konstanz, Konstanz, Germany; 2 University of Münster, Münster, Germany; Tohoku University, JAPAN

## Abstract

The processing of context-dependent expressions has mostly been studied using metaphors, irony or indirect requests. We add to the literature by monitoring the processing of modal particles (MPs) like German *ruhig*. These lexemes are ambiguous between a modal particle reading and a counterpart reading. While the counterpart contributes at-issue or propositional meaning to the sentence and affects truth conditions, the modal particle contributes non-at-issue meaning, expressing speaker attitudes or linking the sentence to the discourse. In the present study we disambiguated the readings via a preceding context and analysed event-related potentials and the oscillatory brain responses to critical sentences with identical surface forms. Our results show that in comparison to their counterparts, modal particles are associated with enhanced P600 amplitudes on the target lexemes and in the spillover region, and with enhanced P200 amplitudes on target lexemes. In addition, modal particles do not show the gamma power enhancement visible for counterparts. We interpret our findings as showing increased processing cost for modal particles due to them contributing to a higher dimension of meaning. The differences in oscillatory brain activity between both readings may reflect the lack of at-issue meaning contribution for modal particles. Our findings are in line with reports of enhanced P600 amplitudes for semantically and pragmatically complex readings in the literature on context-dependent expressions, and open various future ways of research to check the claims of different models on the processing of complex semantic and pragmatic information.

## 1 Introduction

Unambiguous words with a single clearly designated referent are ‘easy’ to process. Some words and phrases however allow multiple interpretations, depending on the discourse context for disambiguation. This provides a challenge, presumably to the parser during online processing, and certainly to models of language comprehension aiming to explain the mechanisms allowing speakers to sucessfully disambiguate words depending on cues that sometimes go beyond even the sentence context. There is a broad range of studies investigating the processing of such context-dependent expressions. Examples of well studied expressions are indirect requests (e.g., [1–5]), irony (e.g., [6–8]), metaphors [9–12], and politeness markers [13,14]. Despite their many obvious differences, these context-dependent expressions are united by the fact that literal and non-literal interpretations are part of everyday speech and are obviously routinely performed by speakers. For most phenomena, studies show that the integration of non-literal meanings and context/discourse information is associated with an increase in processing cost.

While context-dependent expressions have been the topic of much recent psycho- and neurolinguistic research, one has hardly been investigated so far, namely, German modal particles (MPs) like *ruhig*. MPs are a central part of spoken and written language in German. They are short words that all come with a counterpart (CP) in another word class. Both, the MP and the counterpart, contribute different types of meaning. In the sentence *Geh ruhig spielen!* (‘Go play!’), the MP *ruhig* adds a reassuring ‘flavor’ to the sentence (‘Go ahead and play, it’s alright!’), without a straightforward translation into English available. The CP *ruhig* can be translated as ‘quietly’; in this reading, the sentence would mean ‘Go play quietly!’. (A detailed description is given in a following subsection. In the current article, we limit ourselves to a discussion of German modal particles. Generally, modal particles are a prominent feature of a number of languages, certainly among continental West Germanic languages like Dutch, see [15], although existing descriptions tend to be theoretical rather than quantitative.) MPs unite a number of benefits of different context-dependent expressions, making them particularly suitable for investigating the contrast between context-dependent contributions to meaning. Like with politeness morphemes (but unlike e.g. with irony), the difference between the meaning contributions is tied to one specific target lexeme. Like ironical or metaphorical expressions (but unlike e.g. politeness morphemes), they can be compared to their own counterparts in string-identical sentences, thereby providing their own baseline. Unlike literal in contrast to metaphorical usage of individual words, MPs can be assumed to be lexicalized for all speakers of German. Studying MPs allows us to monitor differences between the processing of different, context-dependent meanings (similar to the work on metaphors, irony, and indirect speech acts cited above) with the onset linked to a specific word in the sentence, rather than the expression as a whole. Despite their benefits for experimental pragmatic research, there is currently only a limited number of available studies investigating online processing differences between MP and counterpart readings.

We begin with a short overview of the existing literature on the processing of context-dependent expressions, before providing more background on theoretical and processing accounts of MPs. This will be followed by an outline of the contribution that MPs can make to our understanding of how context-dependent expressions are processed. We then present and discuss the results of an EEG experiment on the processing of an MP and its counterpart in German sentences. The goal of our study is to establish neurolinguistic processing correlates for German MPs and their counterparts and to give an outline of how our findings can inform the wider literature on the processing of context-dependent expressions.

## 2 Background

### 2.1 The processing of context-dependent expressions

Context-dependent expressions include indirect requests, irony, metaphors and politeness markers. The propositional or literal meaning of these expressions can be constructed from the meaning of their parts. In contrast, their non-literal meaning goes beyond their purely propositional, literal meaning. Which meaning is the intended one strongly depends on the context. Despite their many obvious differences, these context-dependent expressions are united by the fact that literal and non-literal interpretation are part of everyday speech and are routinely performed by speakers. Still, the exact mechanism of constructing and processing their non-literal meaning is a matter of ongoing debate. While most evidence points towards mild increases in processing cost for the integration of non-literal relative to literal meaning, there are a number of different suggestions as to the nature and time course of the integration of non-literal meanings. Older, heavily theory-driven approaches like the Standard Pragmatic Model or Hierarchical Model (formulated for the processing of metaphors, [16,17]) consider non-literal readings of context-dependent expressions deviations from normal language processing. According to these approaches, processing is serial, with literal meaning being computed first and non-literal meaning being computed if the literal meaning turns out to be a mismatch in the given context. The opposite approach is taken in the Direct Access Model [18], which holds that in a context triggering non-literal interpretation, the non-literal meaning is accessed directly without necessarily accessing literal interpretation. More nuanced approaches like the Graded Salience Model [19,20] allow for parallel access to all interpretations, with context being one among many factors (like e.g. use frequency). A different line of research is focused less on the time course of access to literal and non-literal readings, and more concerned with questions of the functional relation between the processing of non-literal meaning and other types of information. Two-step models assume that syntactic and semantic information is processed independently of context, and mapped with world knowledge and wider communicative context during later processing stages when the final meaning is computed. If the syntactic-semantic information turns out to be ill-formed, this can block the interpretation of pragmatic and discourse information (also formulated in the Blocking Hypothesis; see [21,22]; [23] for a detailed discussion of possible mechanisms of the functional interplay between structural and pragmatic/discourse information, and a discussion of the alternative single-step model holding that pragmatics is processed independently of syntactic-semantic well-formedness).

None of these models is unambiguously supported by the current psycho- and neurolinguistic literature; still, some generalizations on the processing of context-dependent expressions can be made. Most of the more recent studies show an early influence of pragmatics on sentence processing, argueing against models like the Standard Pragmatic Model that assume a late onset of non-literal meaning processing, and equally against strict interpretations of two-stage models. At the same time, the literal meaning seems to play a role even in non-literal contexts. Many studies find increased processing load for non-literal meanings, which are attributed to additional integration of non-literal information with the wider discourse context, world knowledge and language conventions during unification (see [5,7] for discussion and arguments in favour of one-step approaches, [14] for discussion in favour of a privileged role for syntactic-semantic relative to pragmatic information).

Early findings suggest that indirect requests are not associated with increased processing load relative to direct requests [1], a finding that has held up in later behavioral work [2]; this has been taken to support non-literalist models of illocutionary force. [3] also show no increase in processing cost for conventionalized indirect requests relative to direct requests, but suggest that a conflict between sentence and utterance type (e.g., questions being directives) may lead to a slowdown in reaction times. In contrast, [4] report increased pupil size for the processing of indirect requests relative to statements, suggesting an increase in processing cost for the non-at-issue information contributed by the former, but not the latter. EEG evidence also suggests processing differences between indirect requests and statements disambiguated by a preceding context [5], including late posivities for the former relative to the latter becoming visible on second and third words of the target utterances.

The processing of metaphors relative to literal readings has been associated with amplitude increases for N400 and late positivities [9,10], which are more pronounced for novel literary metaphors than for conventional metaphors [11,12].

Politeness markers like honorifics are another example of context-dependent expressions. Politeness violations can be studied particularly well in languages with morphologically overt politeness markers, since violations can be linked to a specific word position in the sentence. For Mandarin Chinese, [13] show an enhanced N400 in response to overly polite or impolite stimuli (relative to appropriately polite baselines), with impolite conditions followed by late sustained negativities, and overly polite conditions followed by late sustained positivities. The authors proposed that N400 enhancements reflect semantic integration difficulty, and later components reflected different pragmatic rescue strategies; this was taken as showing that the social pragmatics of politeness are processed after syntactic and semantic coherence. In a follow-up study, [14] found that syntactically well-formed sentences with politeness violations elicited an enhanced P200 and a centro-parietally distributed positivity. A combination of syntactic-semantic and politeness violations elicited P600 similar to the ones for syntactic-semantic violations alone. The author concludes that strong violations like those in [13] elicit N400s, while the milder violations in their own study elicit the biphasic P200-P600 pattern. In addition, they conclude that while pragmatic information is processed quickly (visible in the P200 enhancement for politeness violations), syntactic/semantic processing has functional primacy over politeness processing, meaning that politeness violations will not be processed when syntactic/semantic violations make the sentence ill-formed.

Yet another comparatively well-researched contrast between literal and non-literal meanings is provided by irony. For identical sentences disambiguated between literal and ironic interpretations by a preceding context, the usual findings are increases in P600 amplitude [6,7]. [24] report enhanced P200 and P600 amplitudes for ironic relative to literal conditions, and oscillatory differences in the N400 and P600 time windows for syntactic and pragmatic effects (stronger decrease in alpha activity for pragmatic than for syntactic effects in the N400 time window; stronger increase in theta activity for syntactic than for pragmatic effects in the P600 time window). They interpret their findings as reflecting the early recognition of non-literal meanings, followed by increased processing load for the integration of linguistic and contextual information. Interestingly, P600 effects are less extensive and of lower amplitude than those for syntactic violations; effects of irony and syntactic violations seem to have an additive effect on P600 amplitude (in contrast to the ones reported for politeness violations in [14], suggesting that syntactic/semantic information may not generally take primacy over pragmatic processing). Similar P200-P600 enhancements for ironic relative to literal conditions were reported by [8], with the P600 amplitude related to participants’ interpretation of the stimuli. [7] also report differences between literal and ironic conditions in oscillatory activity, with gamma band activity increases for ironic conditions between 280 and 400 ms. The authors interpret these findings as showing that different streams of information are integrated early during the processing of non-literal expressions.

Taken together, the wider literature on the processing of context-dependent expressions paints the following picture: (a) If supported by a preceding context, non-literal contributions to meaning are recognized and integrated quickly. (b) For most phenomena, integration of non-literal meanings and context/discourse information is associated with an increase in processing cost, most often visible in P600/LPC enhancements, and occasionally in N400 or P200 enhancements. (c) The extent and amplitude of this P600 enhancement is smaller than that for syntactic/semantic violations, suggesting a relatively mild increase in processing cost.

Current research on the processing of context-dependent expressions hinges on a number of issues, among them the choice of an appropriate baseline, and picking conventionalized or original non-literal expressions. Furthermore, more recent models of processing non-literal meaning are concerned with the timing of access to literal and non-literal meaning. While the processing of modal particles is interesting in its own right (given their ubiquity in languages like German or Dutch), they additionally unite a number of beneficial properties for researching context-dependent expressions. Establishing their processing correlates thus opens possibilities to contribute new insights to the wider discussion on context-dependent processing. In the following, we give a short theoretical description of their linguistic properties before presenting the limited available psycholinguistic research.

### 2.2 Theoretical accounts of modal particles

Modal particles (MPs) are highly context dependent for at least two reasons. First, they are ambiguous, as each MP has a counterpart in another word class, such as adverbs, focus particles, or conjunctions. Thus, when being confronted with a lexeme that functions as an MP, the hearer or reader has to figure out the intended meaning. Second, MPs make a very specific meaning contribution that has been labeled *non-at-issue* [25]. The meaning of a sentence can be divided into meaning components that are at-issue (AI) and non-at-issue (NAI). While AI refers to what [16] calls ‘what is said’, NAI meanings can be seen as side-comments, not contributing to ‘what is said’ [26]. An example of NAI meanings are conventional implicatures. They are encoded in specific lexical items and constructions in a more or less idiosyncratic fashion [26]. Sentences hosting MPs feature AI content to which the MP independently adds NAI content. As a counterpart, however, it features AI content. Thus, there is one lexical item that contributes, dependent on the context, two different meanings, namely an AI and a NAI meaning.

Take, for example, the lexeme *ruhig*. The translation of its counterpart reading is ‘quietly’, ‘calmly’, ‘steadily’ (see (1)). As a counterpart, *ruhig* contributes to the AI meaning of the sentence by describing the manner in which the playing event has to be carried out. It has a translation equivalent (‘quietly’) and it is not restricted to a specific formal environment.

(1) Geh **ruhig**    spielen!
  go    RUHIG.cp play
  ‘Go play **quietly**!’ [You may go and play, but don’t make any noise, your grandmother is asleep next door.]

As an MP, *ruhig* is referred to as ‘the friendly particle’ [27] It does not influence the AI meaning of the sentence hosting the MP, but adds a NAI meaning. According to [27], when *ruhig* is added to a sentence, mostly a flavor of reassurance is obtained. It has no translation equivalent, but it can be paraphrased by ‘no worries’. Functionally, it is restricted to sentences that are used mostly for permissions or recommendations. In sentences that are used for commands, for instance, the MP *ruhig* is excluded. Formally, *ruhig* is restricted to imperatives or declaratives containing the possibility modal *can*. Crucially, sentences containing the necessity modal *must* cannot host the MP *ruhig* [28] (see (2)). Co-occurring with the necessity modal *must*, *ruhig* can only be interpreted as the counterpart.

(2) Du kannst/ #musst **ruhig**    spielen!
  you can    must    RUHIG.mp play
  ‘You may go play (no worries)!’ [Go ahead, it’s alright.]

Sentences containing MPs can be globally ambiguous. Example (1), for instance, can have two different meanings, depending on the reading of the ambiguous lexeme (triggered by the context) (see (3)). As a counterpart, it directly influences the compositional meaning of the sentence by adding AI content (i.e., the manner in which the action has to be carried out). As an MP, it does not influence the AI meaning of the sentence, but adds NAI meaning (i.e., the attitude of the speaker towards the future course of events).

(3) Geh **ruhig**   spielen!
  go RUHIG play
  CP: ‘Go play **quietly**!’ [You may go and play, but don’t make any noise, your grandmother is asleep next door.]
  MP: ‘Go play (no worries!)!’ [Go ahead, it’s alright.]

The differences between the AI meaning of the host sentence and the counterpart on the one hand, and the NAI meaning conveyed by the MP on the other hand, is modeled by the hybrid-semantics framework by [29]. His approach is primarily based on the semantic tools developed by [30] for dealing with conventional implicatures and on [31]’s remarks on how expressions that contribute use-conditional rather than truth-conditional content might be integrated into a formal framework. The framework is a multi-dimensional approach, featuring multiple dimensions to capture the AI and NAI meanings. Applied to our example (3), the MP *ruhig* takes the propositional argument *go play*, returns it in the first dimension, and expresses its specific attitude towards the propositional argument in the second dimension. After expressing this attitude in the second dimension, the MP *ruhig* starts with a trivial third dimension, where AI- and NAI-content, or different NAI-contents, interact (see [29]). This third dimension is especially interesting for MPs, as they are sentence-mood dependent (e.g., the MP *ruhig* is only felicitous in imperatives, but not in declaratives, except when they contain a possibility modal). That is, for a sentence to be felicitously uttered, the attitudes expressed by sentence mood and the contribution conveyed by MPs both have to hold [29], and this interaction is assumed to take place in the third dimension.

Interestingly, due to the different meaning dimensions, the MP *ruhig* and the counterpart *ruhig* can recur within the same clause, as shown in (4). This is only possible when both lexemes feature different meaning contributions.

(4) Du kannst **ruhig**   **ruhig**   spielen.
  you can   RUHIG.mp RUHIG.cp play
  CP: ‘Go play **quietly** (no worries)!’ [Go ahead, it’s alright. You may go and play, but don’t make any noise, your grandmother is asleep next door.]

Taken together, there are ambiguous lexemes that convey, dependent on the context, two different meanings: An AI meaning that influences the propositional meaning of the sentence, and an NAI meaning that does not influence the propositional meaning of the sentence, but mostly expresses a specific speaker attitude towards the sentence. To date, there has only been little research on the processing of these ambiguous lexemes and their different meaning contributions, as outlined in the following.

### 2.3 Processing modal particles

**Behavioural background.**

As outlined in the introduction, MPs unite a number of benefits of different context-dependent expressions, making them particularly suitable for investigating the contrast between context-dependent contributions to meaning. Since all MPs come with CP counterparts (adding AI meaning), they can be compared to identical lexemes (with different readings) in string-identical sentences, effectively making their own baseline and avoiding lexical confounds. The difference between AI and NAI contributions to meaning is tied to one specific target lexeme that comes in the same position in these sentences; this makes them particularly suitable for investigating their online processing in word-by-word presentation paradigms. Finally, MPs can be assumed to be lexicalized and well-known to all native speakers of German, so the processing of NAI contributions to meaning can be seperated from the processing of novel or creative language use. (An overview of the similarities and differences to other context-dependent expressions, as outlined in the introduction, is given in Table 1.)

**Table 1:** Overview of the properties of different context-dependent expressions.

context-dependent expression	property		
	one target lexeme	identical counterpart	conventionalized/lexicalized
MPs	✓	✓	✓
irony	✗	✓	✗
indirect request	✗	✓	✓
politeness morphemes	✓	✗	✓
metaphors	depends	✓	depends

‘One target lexeme’ refers to whether the difference in meaning contribution is tied to one particular word or morpheme. ‘Identical counterpart’ indicates if there are two string-identical sentences that make different contributions to meaning depending on the context, while being well-formed in both contexts. ‘Conventionalized/lexicalized’ refers to whether the context-dependent expression can be assumed to be well-known to speakers or even part of their lexicon and is used in day-to-day language.

Despite their benefits for experimental pragmatic research, there is currently only a limited number of available studies investigating on-line processing differences between MP and counterpart readings.

[32] and [33] show that MPs lead to increased reading times relative to their counterparts in string-identical sentences when readings are disambiguated via a preceding context, indicating that processing the NAI content added by the semantically ‘weak’ MP is linked to increased processing costs. [32] and [33] established usage frequencies and investigated the processing of twelve different lexemes featuring an MP reading (NAI meaning) and a counterpart reading (AI meaning). These lexemes were *auch* (‘also’), *bloß* (‘only’), *doch* (‘nevertheless’, ‘but’), *eben* (‘just’, ‘flat’), *einfach* (‘easy’, ‘simple’), *erst* (‘first’), *gleich* (‘immediately’, ‘same’), *nur* (‘only’), *ruhig* (‘quiet’), *schon* (‘already’), *vielleicht* (‘probably’, ‘perhaps’), and *wohl* (‘possibly’, ‘good/well’) (translations of counterparts in parentheses).

For these twelve lexemes, [32] and [33] collected meaning frequency data in order to determine the dominant and the subordinate meaning of each. Based on 13.100 sentences from the DWDS corpus [34], the authors assessed the meaning of each lexeme by taking into account the surrounding discourse context. Their findings show that relative proportions of MP and counterpart readings differ considerably, ranging from 1% MP readings for *gleich* to 80% MP readings for *wohl*.

In a series of self-paced reading time studies, they investigated the processing of MP and counterpart readings for ten target lexemes (*auch, bloß, doch, eben, einfach, gleich, nur, ruhig, schon*, and *vielleicht*) in identical target sentences. The readings were disambiguated via a context. Contexts either preceded or followed the target clauses. The results showed that in conditions with preceding contexts, MP readings were associated with increased reading times relative to their counterpart readings, beginning on the second word following the critical word. In addition, the frequency bias of different readings influenced reading times, interacting with the position of the context and the reading triggered by the context on the third word after the critical word (while lexeme frequency did not show an influence on reading times). These findings suggest that processing the NAI content of MP readings is more costly than processing the AI content of the counterparts, and that this increase in processing cost for MP readings cannot be reduced to effect of frequency bias or lexical frequencies. The authors take this to support the idea of two-dimensional meaning representation of NAI and AI content.

**Neurolinguistic background: ERPs.**

To date, there is no neurolinguistic study offering a one-on-one comparison of MPs and counterparts. This is not surprising given the host of different factors that need to be controlled in these comparisons, and the fact that many languages which have been thoroughly researched in neurolinguistics (like English) do not prominently feature MPs.

The closest neurolinguistic topic is the processing of question-sensitive discourse particles, described in [35] and [36]. Question-sensitive discourse particles (QDiPs) like *denn* are special MPs that modify the illocutionary force of questions, and are subject to a number of interacting licensing constraints. In the cited studies, the authors compare the processing of QDiPs relative to a non-QDiP baseline, manipulating the fit of the QDiP conditions with different licensing constraints. In the context of our current study, the relevant results are the following: (a) Relative to non-QDiPs, QDiPs elicit enhanced P600 amplitudes. (b) This increase in P600 amplitudes is relatively short-lived for well-licensed QDiPs, while it continues until the end of word presentation for unlicensed QDIPs (i.e., in grammatically ill-formed structures). (c) A mild increase in N400 amplitudes for QDiPs relative to the baseline in some comparisons, and (d) An increase in theta power for non-QDiP baselines relative to QDiPs [36].

The authors speculate that the increase in P600 for QDiPs relative to the baseline reflects the increased demands for their syntactic, semantic and discourse integration. The fact that QDiPs show less theta enhancement than non-QDiP baselines is taken to reflect the fact that unlike the baseline lexemes, they belong to a closed word class (see [37–39] for a discussion) and do not contribute semantic AI meaning that would lead to increased lexical activity. – However, these findings on a contrast between AI and NAI content come from a comparison of two different lexemes in structures with different syntactic and semantic processing demands. Therefore, they only allow a limited amount of prediction for the processing correlates of MPs relative to their counterparts in well-formed structures.

**Summary background processing.**

Taken together, the following picture about the processing of MPs emerges:

- Processing costs for MPs are higher than those for their counterparts. Self-paced reading times show an increase in processing load on the first word after the MP, and continuing for at least three words (i.e., until the end of the sentence).
- While NAI meaning (MPs) is related to increased processing costs relative to AI meaning (counterparts), the relative frequency or which reading is the dominant one also affects processing measures.
- Currently, there are no known neurolinguistic correlates of modal particle processing. The few existing neurolinguistic studies on German particle processing are focused on issues of complex syntactic licensing processes and do not directly compare MPs to their counterparts.

**Research goals and predictions.**

This summary shows that the first findings on the processing of German modal particles are encouraging, suggesting a close link between theoretical description and measurable processing cost (i.e., increased reaction times for conditions associated with constructing complex NAI meaning relative to AI meaning).

Our research goal is to establish the neurolinguistic correlates for processing MPs relative to their counterparts. We will monitor differences both in the event-related and the time-frequency domain, adding quantitative and qualitative nuance to the more coarse-grained findings from earlier behavioral studies.

Doing so will add important detail to our knowledge about the quality and timing of constructing different types of meaning, allowing us to interpret our findings with respect to ERP and oscillatory correlates with established links to language processing. In addition, it will allow us to replicate the earlier behavioral findings using a different methodology, lending additional support to the proposed theoretical distinction between AI and NAI meaning and its relevance for language processing. This is relevant for a better understanding of modal particle processing (and thus a central part of communication in many languages). It also contributes to the wider literature on context-dependent expressions, adding knowledge on a new phenomenon with a number of beneficial properties for experimental linguistic research (see our discussion above), and opening many possibilities for future research, e.g. on issues of timing literal meaning vs. context-dependent meaning.

We will pursue this research goal in a similar way to [32], namely by comparing between a lexeme that has an MP and a counterpart reading, and that occurs in string-identical sentences. The different readings will be disambiguated by a preceding context clause. To minimize the influence of meaning frequency bias established in [32,33], we will only consider one lexeme that has roughly similar frequencies of MP and counterpart readings (see below for details).

Based on the behavioral findings reported in [32,33], we assume an increase in processing cost for MP relative to counterpart readings. This increase in processing cost will affect words in the spillover region following the target lexemes; but early effects on the target lexeme proper may also become visible in the more time-sensitive EEG measurements.

Based on the literature reviewed above, we assume that pragmatic and non-literal information is processed quickly. At this point, we do not make predictions on the functional relation between syntactic-semantic and pragmatic contributions to meaning, i.e., we are not aiming to find out whether syntactic/semantic violations do or do not block the processing of the NAI meaning provided by MPs. Tackling related research questions will become possible once the processing correlates of MP processing have been established; see also our discussion. Based on the findings reported in the neurolinguistic literature on the processing of context-dependent expressions and on the processing of discourse particles (reviewed in detail above), we assume that MP readings will most likely be associated with higher P600 amplitudes than counterpart readings, reflecting increased workload for the construction of non-at-issue meaning and unification demands (see [14] for mild politeness violations, and [6,7,24] for irony). Since the preceding context disambiguates the readings and modal particles are highly conventionalized, we expect that their NAI meaning contribution will be recognized and processed quickly; this may lead to a P200 enhancement relative to CP conditions (in line with P200 enhancements reported for other non-literal expressions, see [13,14] for politeness markers, and [8,24] for irony). Finally, expecting an N400 enhancement for MP readings relative to CP readings is possible, but less likely than the preceding expectations. While there have been some reports of N400 enhancements for literal relative to non-literal meaning, these studies differ from our own in that they involve increased demands for semantic processing, either by strong violations (see [13] for politeness violations), or creative use of language as in metaphors [10–12]. Predictions for oscillatory activity are more difficult to formulate, given that the literature on oscillatory activity during language processing is younger and is evolving rapidly. Increased processing cost for semantic-pragmatic unification for MPs relative to counterparts might be reflected in increased beta and gamma band activity [38], and possibly theta-band activity [7], for MPs. (Consider however, that the more recent literature links integration mainly to high gamma-band activity, while low gamma band activity is linked to reactivation of stored information during sentence processing; see [40] for a discussion. Since our data required filtering at 40 Hz, our analysis was restricted to frequencies up to this threshold, preventing us from drawing any conclusions about high gamma band processes.)

At the same time, we could expect oscillatory activity to reflect increased lexical-semantic processing for the AI information associated with counterparts, leading to higher activity in the alpha band for counterpart than MP due to lexical retrieval ([41]; [42] for a review). While some of the literature would also lead us to expect increased theta band activity for counterparts relative to MPs [37,38,43,44], this would run counter to expected increases in pragmatic processing cost for MPs relative to counterparts, making predictions for this frequency band particularly difficult (see also [36] for a discussion). Similar expectations hold for the gamma band, where increases in activity have also been linked to the processing of bottom-up semantic information [45]. – Given the difficulty of formulating clear predictions, we will approach the analysis of oscillatory activity in an exploratory spirit, and will discuss our findings in the context of this emerging subfield of neurolinguistics.

We test these predictions in an EEG experiment described below.

## 3 Materials and methods

### 3.1 Language material and stimulus presentation

**Critical items.**

We chose the lexeme *ruhig*, described in detail above, as the ambiguous lexeme for our stimuli. *Ruhig* is the most suitable lexeme for our purposes: It is one of the lexemes with the most balanced distribution of MP and counterpart (CP) readings (32.6% MP meaning frequency), and with the strongest semantic contrast between the MP and the CP reading. This enables us to disambiguate the different meanings via a preceding context.

We constructed 30 items with *ruhig*, which come in two conditions each. All items consist of a context sentence ending in a full stop, followed by the critical sentence containing the target lexeme. The purpose of the context sentence is to provide a tiny discourse environment and, more importantly, to disambiguate the target lexeme towards the CP or MP reading. Context sentences begin with two names (as in a play script) suggesting that the following two sentences are part of an ongoing dialogue. Critical sentences always follow the pattern *You - modal-verb - DP - target lexeme - preposition - DP - infinitive*. The modal verb in the CP condition is the necessity modal *musst* (‘must’), the modal verb in the MP condition is the possibility modal *kannst* (‘can’). This makes the respective readings sound natural and further formally disambiguates between readings (see [28]). Apart from the modal verbs (i.e., beginning on the second word before the target lexeme), critical sentences in both conditions are string-identical. A typical example of an item in both conditions is given in Example 1, with Example 1 (A) illustrating the CP condition and Example 1 (B) illustrating the MP condition.

**Example 1. A stimulus pair illustrating both conditions with both readings of *ruhig*.** CP refers to the counterpart reading, MP refers to the modal particle reading.

CP-triggering context:

*Pia zu Leni: Es ist wichtig, dass der Wirkstoff sich gut verteilen kann.*

‘Pia to Leni: It is important for the active ingredient to disperse well.’

(A) CP-ruhig:
  Du musst die Tropfen ruhig   ins   Auge machen.
  You must the drops   CP-RUHIG into.the eye   put
  ‘You have to apply the eye drops calmly.’

MP-triggering context:

*Pia zu Leni: Ich habe mir den Beipackzettel durchgelesen.*

‘Pia to Leni: I read the package insert.’

(A) MP-ruhig:
  Du kannst die Tropfen ruhig   ins   Auge machen.
  You can   the drops MP-RUHIG into.the eye put
  ‘[It’s alright], you can apply the eye drops.’

**Fillers.**

Critical sentences were interspersed with 120 filler items to provide variety and avoid priming effects. All filler sentences are similar to the critical sentences in that they begin with a context sentence presented as a whole, followed by a second sentence presented word by word. Filler items came in two varieties: Half of the filler items contained the lexeme *schon* in the second clause, which like *ruhig* is ambiguous between MP and CP readings, preceded by different context clauses. These fillers were added to avoid having *ruhig* as the only MP/CP-ambiguous lexeme. While different readings of *schon* are more challenging to disambiguate in our type of stimulus material, we chose *schon* for the fillers because just like *ruhig*, the frequencies of available readings are relatively balanced (34.7% MP meaning frequency), compared to other candidate lexemes (like *vielleicht* with 2% MP meaning frequency or *wohl* with 80% MP meaning frequency). The goal was to avoid a bias towards the MP or CP reading in the critical stimuli. An example of a typical filler item with *schon* is *Luca zu Fatima: Ich plane noch die letzten Einzelheiten und du kannst die Sache schon ins Rollen bringen.* (‘Luca to Fatima: I will take care of the final details and you can already get things started’).

The other 60 filler items began with a clause describing someone making a statement, and ended with the content of the statement. An example of a typical filler is ‘ *Ingo hat mir gestern erzählt, dass er eine Wette gewonnen hat.* (‘Ingo told me yesterday that he won a bet.’). Filler items belonged to one of five ‘pseudo-quartets’ sharing the first part of the sentence including the connective *dass*, but differing in the latter part. This was done to also offer some stimuli where the first, rather than the second, part of the stimulus remained identical.

In the total stimulus list, 1/3 of stimuli were critical items with *ruhig*, and 2/3 were filler items. All items included in the EEG experiment were well-formed, containing no violations.

To provide participants with a task, we asked for comprehension questions after 1/4 of the sentences (16 after critical conditions, 19 after fillers), balanced across conditions, amounting to 45 questions in total. Questions did not call attention on differences between CP- and MP-readings.

**Pretest: Semantic fit.**

To see if the target sentences were a good semantic fit with the preceding context, we ran a pretest with eight participants, using the stimuli containing potentially ambiguous lexemes. The sentences were presented together with their context. Participants were asked to rate the felicity of the critical sentence (when following the context sentence) on a 5-point Likert scale, with 5 indicating the highest felicity rating and 1 the lowest (i.e., a bad fit). Since rating the felicity of a stimulus set where all items are supposed to be felicitous is a difficult task, we added pretest filler items that were clearly a bad fit, which were similar to the critical items, contained the MP, and had the same structure as the MP and CP triggering context conditions. An example of a pretest filler similar to the CP condition is *Dina zu Christian: Ich möchte in den Zirkus gehen. Du musst das Flugzeug ruhig auf die Landebahn bringen.* (‘Dina to Christian: I want to go to the circus. Steady as you get the plane on the landing strip.’) - while both sentences are grammatical, they don’t make for a coherent or natural mini-discourse. The experiment contained all 30 experimental pairs with *ruhig*, all 30 filler pairs with *schon*, 10 bad pretest fillers containing *ruhig* and ten bad pretest fillers containing *schon*. Mean ratings were markedly lower for the infelicitous pretest fillers (mean = 1.21, s.e. = .25) than for the critical conditions (CP: mean = 3.51, s.e. = 1.34; MP: mean = 4.33, s.d. = 1.08). Planned comparisons revealed a small but statistically significant difference between MP and CP conditions (*t*(7) = 2.7, *p* < .05). Since both CP and MP readings of *ruhig* are well within the acceptable range and the numerical difference between felicity ratings for both conditions is small, we decided to accept the difference.

**Stimulus presentation in the EEG experiment.**

Stimuli were presented on a black screen in white letters (Arial, 44 pt). Presentation began with a fixation cross for 500 ms, followed by a 200 ms blank screen. Then, context clauses were presented as a whole for an unlimited time until participants pressed a button to indicate they had read and understood the context. After participants had pressed the button, there was a blank screen for 200 ms, followed by the presentation of the critical sentence. The critical sentences were presented word by word. Each word was presented in the center of the screen for 600 ms, followed by a 200 ms blank screen. After the last word of the sentence, there was a 1000 ms blank screen.

After 1/4 of all sentences, a comprehension question was asked to give participants a task and monitor attention. Questions did not direct attention towards reading differences between CP and MP conditions. 16 questions came after critical stimuli (eight questions for CP conditions, eight questions for MP conditions, four of each with the correct answers YES or NO respectively), 29 questions came after filler sentences (15 with the correct answer YES, and 14 with the correct answer NO). Questions were presented in a green font. There was no time-out for responses, and no feedback to the answers was given during the EEG experiment. After participants had given their response, the question disappeared, followed by a 1000 ms blank screen. All participants saw all stimuli.

### 3.2 Participants

44 participants were tested at the Neurolinguistics lab of the University of Konstanz. Participants were recruited between March 15th 2023 to 15th November 2023 using the Konstanz University SONA systems participant database. Participant recruitment, consent, experimental procedure and data handling and storage were reviewed by the Internal Review Board of the University of Konstanz, and were found to be in line with the ethics regulations of the University of Konstanz for ethical experimentation and data storage, the Declaration of Helsinki as developed by the World Medical Association, and the relevant national and international laws and regulations, as per IRB statement 05/2021. All participants received oral and then written instructions and explanations about the procedure and gave written and informed consent. Participants were informed that they could stop the experiment at any time without giving an explanation and without facing negative consequences. All participants completed the experimental session.

Before data analysis, two participants were removed due to movement artifacts and excessive alpha waves, leading to low data quality. Of the remaining 42 participants, 26 were female and 16 were male. Mean age was 23.4 years (s.d. = 3.3 years, min = 19 years, max = 33 years). Mean answer accuracy in critical items was 95% (s.d. = 5.2%, minimum accuracy = 81%, maximum accuracy = 100 %).

### 3.3 Procedure

Participants were comfortably seated in a sound-attenuated room in front of a monitor at approximately two meters distance. They were asked to avoid excessive eye and body movements during the EEG recording and to avoid crossing their arms and legs. The EEG was recorded with BrainVision Recorder (version 1.24.0001, Brain Products GmbH), with 64 EEG actiCAP slim electrodes, attached to an elastic cap with actiCAP SNAP holders and connected to BrainAmp DC amplifiers. The electrode arrangement was based on the equidistant M43-V1 layout as provided by Easycap GmbH. Horizontal and vertical eye movements were registered by four EOG Ag/AgCl sintered passive ring electrodes, connected to BrainAmp ExG bipolar amplifier. Data were recorded in the frequency range 0.016–250 Hz. Impedance values below 20 kOhm were accepted. The signal was digitized with a sampling rate of 500 Hz. Stimuli were presented and responses were recorded using the Presentation software by Neurobehavioral Systems Inc. (version 20.2). Each session lasted about 40 minutes, and was interspersed by 3 breaks.

### 3.4 Data preparation and analysis

**ERP data preparation and analysis.**

Data were processed using the Brain Vision Analyzer 2 software (Brain Products, Gilching). We began data preparation with filtering (low cutoff 0.5 Hz, high cutoff 40 Hz, 50 Hz notch filter), followed by a manual raw data inspection, an ICA blink correction, and if necessary and topographic interpolation via spherical splines. After interpolation, all electrodes were re-referenced to average reference. An Automatic Raw Data Inspection was performed for the re-referenced data (maximal allowed voltage step: 50 μV/ms; maximal allowed difference: 100 μV/200 ms; minimal/maximal allowed amplitudes 200 μV/−200 μV; lowest allowed activity: 0.5 μV/100 ms).

Segments were cut so that they included the target lexeme *ruhig* and the two following words (*ruhig*+1, a preposition, and *ruhig*+2, a noun). Each segment begins 200 ms before the onset of the target lexeme and ends 2400 ms after the onset of the target lexeme, i.e., 800 ms after the presentation of word x+2. We chose to cut longer segments because the behavioral literature on similar stimuli [32] reported spillover effects lasting for several words after the target lexeme. Choosing the target lexeme and the two following words allowed us to monitor long-lasting differences between conditions, while still working with a reasonably high number of segments per condition and without including the last word of the sentence that might have added wrap-up effects to the data. Following a baseline correction for each segment (200 ms baseline), average curves were calculated for each participant and condition. The mean number of segments in participant averages was 28 segments. The minimum number of segments in a participant average for a single condition was 21.

Grand averages were used to visually identify time windows of interest for the comparison of MP and CP conditions, guided by the hypotheses outlined above (i.e., focusing on P200, N400 and P600 time windows). For each time window of interest, mean amplitudes for each participant were exported.

Data were analysed with spatial downsampling to a 25 electrode subset consisting of electrodes Fz, FCz, Cz, CPz, Pz, F1, FC1, CP1, P1, PO3, F2, FC2, CP2, P2, PO4, F7, FC5, C5, P5, PO7, F8, FC6, C6, P6, PO8. These electrodes were chosen to allow a good resolution of the skull areas relevant for the expected EEG components, in particular, the N400 and the P600. Electrodes in both subsets were parametrized in five medial-lateral and five anterior-posterior regions, added as two topographical factors to the subsequent analyses. This led to the factor medial-lateral (ml for brevity, levels *left* (F7, FC5, C5, P5, PO7), *left-medial* (F1, FC1, CP1, P1, PO3), *medial* (Fz, FCz, Cz, CPz, Pz), *right-medial* (F2, FC2, CP2, P2, PO4), *right* (F8, FC6, C6, P6, PO8), anterior-posterior (ap for brevity, levels *anterior* (Fz, F1, F2, F7, F8), *anterior-central* FCz, FC1, FC2, FC5, FC6), *central* (Cz, CP1, CP2, C5, C6), *posterior-central* CPz, P1, P2, P5, P6), *posterior* (Pz, PO3, PO4, PO7, PO8), and reading (levels *CP, MP*). The influence of the main effects and interactions of these factors on mean amplitudes were investigated in a 5x5x2 ANOVA.

**Time-frequency analysis.**

The time-frequency decomposition was conducted on epochs ranging from -2.4 seconds to 2.4 seconds using the MATLAB-based FieldTrip toolbox [46]. Due to the necessity of filtering our data at 40 Hz, our examination was limited to frequencies up to 40 Hz. We applied Morlet wavelets to individual trial data, stepping in 10-millisecond increments from -2.4 to 2.4 seconds. The width of the frequency-specific window varied linearly, increasing from 2 to 6 cycles for frequencies ranging from 1 to 40 Hz. The resulting output was a complex Fourier spectrum. Baseline correction was applied for a range from -800 to -300 ms relative to the onset of the critical word presentation. To evaluate the statistical significance of differences between various readings (adverb vs. particle) for *ruhig* (i.e., *ruhig* as an adverb of manner vs. *ruhig* as a particle), a multi-level statistical approach was applied. Initially, we conducted independent samples regression coefficient *t*-tests for the entire epoch. Each condition (MP and CP) was compared against one another. The resulting *t*-values underwent *z*-transformation. Following this, Monte Carlo non-parametric cluster-based permutation tests were performed with 1000 randomizations, focusing on three time windows after the onset of the critical word: the target lexeme *ruhig* (0-800 ms), and the two following words *ruhig+1* (800-1600 ms) and *ruhig*+2 (1600-2400 ms) [46].

## 4 Results

### 4.1 ERP results

We identified two time windows with differing curves on *ruhig*, two time windows on word *ruhig*+1, and one time window on word *ruhig*+2. We describe time windows by referring to their start and end time relative to the onset of the target lexeme *ruhig*. For words in the spillover region, we additionally provide the start and end time relative to the onset of the respective word in parentheses; this will allow for a more transparent link between time windows and potential ERP components. ERP results are described for each identified time window in turn. All reported differences are statistically significant unless indicated otherwise. Interactions involving the factor reading are resolved hierarchically.

#### 4.1.1 *ruhig.*

**Fig 1:**
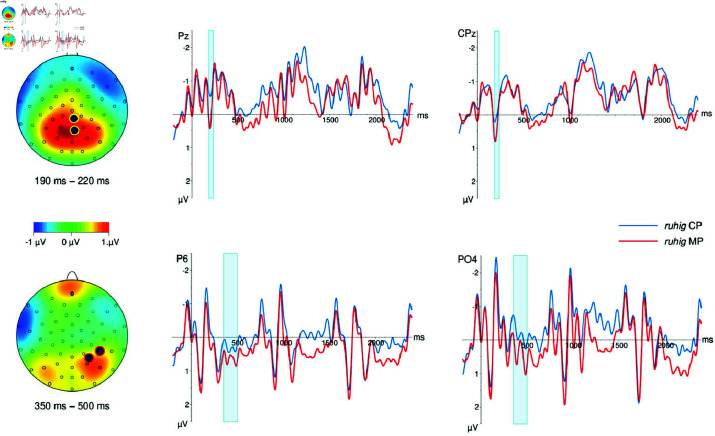
Ruhig, voltage difference maps and ERP curves for selected electrodes. The upper half of the figure depicts the early time window from 190–220 ms, the lower half depicts the later time window from 350–500 ms. The position of the selected electrodes is marked on the voltage difference maps with circles in contrasting colors to their surroundings. Time windows are marked in light blue in ERP curves.

Presentation of the target lexeme *ruhig* went from 0 to 800 ms post target onset (including the ISI). We identified two time windows, an early time window lasting from 190–220 ms post word onset, and a later time window lasting from 350–500 ms post word onset. Waveforms on the critical word *ruhig* and voltage different maps for the time windows are depicted in Fig 1.

***ruhig*, early time window, 190–220 ms.**

Descriptively speaking, relative to the CP baseline waveforms for MP readings were more positive-going at central and posterior electrodes, and more negative-going at marginal electrodes (around the skull).

There was a statistically significant main effect of reading (*F*(1,41) = 7.91, *p* < .01, and marginally significant interactions of reading and medial-lateral (*F*(4,164) = 2.78, *p* < .07, HFε = .53) and reading and anterior-posterior (*F*(4,164) = 3.13, *p* < .07, HFε = .36). The main effect of reading was significant in anterior-posterior regions *posterior* (*F*(1,41)=7.10, *p* < .05), *posterior-central F*(1,41) = 8.00, *p* < .01), marginal in *central* (*F*(1,41) = 4.03, *p* < .06). The main effect of reading was significant in medial-lateral regions *right-medial* (*F*(1,41) = 4.91, *p* < .05), *medial* (*F*(1,41) = 6.84, *p* < .05) and *left-medial* (*F*(1,41) = 7.73, *p* < .01).

***ruhig*, later time window, 350–500 ms.**

Descriptively speaking, relative to the CP baseline waveforms for MP readings were more positive-going at posterior sites, and more negative-going at marginal and some frontal and fronto-central sites. The posterior positivity was a broad positive shift becoming visible shortly after 300 ms that became less pronounced after about 500 ms for most electrodes, while it kept going until the end of the presentation of the target lexeme for others. There was a significant interaction of reading and anterior-posterior (*F*(4,164) = 3.67, *p* < .05, HFε = .40). reading was significant in anterior-posterior region *posterior* (*F*(1,41) = 6.14, *p* < .05).

#### 4.1.2 *ruhig*+1.

**Fig 2:**
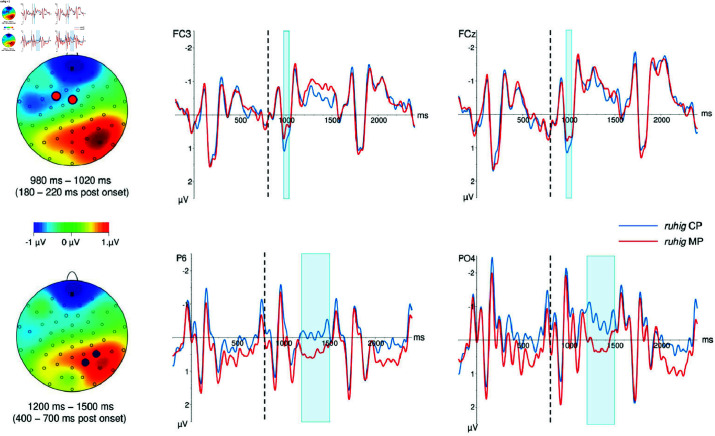
Ruhig+1, voltage difference maps and ERP curves for selected electrodes. The upper half of the figure depicts the early time window from 180–220 ms post word onset, the lower half depicts the later time window from 400–700 ms post word onset. The position of the selected electrodes is marked on the voltage difference maps in contrasting colors to their surroundings. Word onset is marked with a dashed line; the end of the baseline and onset of the target word *ruhig* is marked with a solid line. Time windows are marked in light blue in the ERP curves.

Presentation of the first word after the target lexeme, *ruhig*+1, went from 800 to 1600 ms post target onset. We identified two time windows, an earlier one 180 to 220 ms after word onset, and another one from 400 to 700 ms after word onset. Waveforms on the word *ruhig*+1 and voltage difference maps for the time windows are depicted in Fig 2.

***ruhig*+1, early time window, 180–220 ms post word onset (980–1020 ms post target onset).**

Descriptively, waveforms were more positive-going for MP than for CP at anterior sites, and more negative-going for MP than for CP at posterior sites.

There was a statistically significant interaction of reading and anterior-posterior (*F*(4,164) = 6.71, *p* < .01, HFε = .36). reading was significant in anterior-posterior regions *posterior* (*F*(1,41) = 9.45, *p* < 01) and *anterior* (*F*(1,41) = 5.19, *p* < .05), and marginal in *posterior-central* (*F*(1,41) = 3.92), *p* < .06).

***ruhig*+1, later time window, 400–700 ms post word onset (1200–1500 post target onset).**

Descriptively, waveforms were more positive-going for MP than for CP at posterior sites, particularly on the right side of the scalp. There was an interaction reading and anterior-posterior (*F*(4,164) = 6.03, *p* < .01, HFε = .38). reading was significant in anterior-posterior regions *posterior-central* (*F*(1,41) = 6.02, *p* < .05) and *posterior* (*F*(1,41) = 8.14, *p* < .01).

#### 4.1.3 *ruhig*+2.

**Fig 3:**
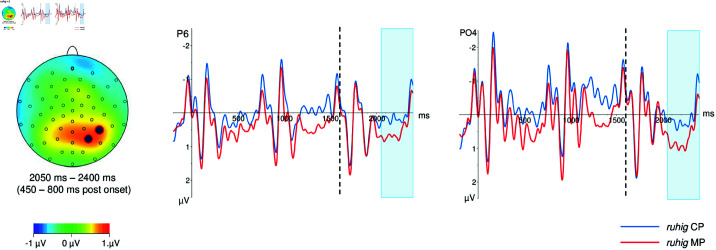
Ruhig+2, voltage difference map and ERP curves for selected electrodes in the time window from 450–800 ms post word onset. The position of the selected electrodes is marked on the voltage difference map in contrasting colors to their surroundings. Word onset is marked with a dashed line; the end of the baseline and onset of the target word *ruhig* is marked with a solid line. The time window is marked in light blue in the ERP curves.

Presentation of the second word after the target lexeme, *ruhig*+2, went on from 1600 to 2400 ms after target onset. We identified one time window, lasting from 450 to 800 ms after the onset of word *ruhig*+2. Unlike in the two preceding words, no striking differences between readings were visible at earlier times (i.e., around 200 ms after word onset). Waveforms on the word *ruhig*+2 and voltage difference maps for the time windwos are depicted in Fig 3.

***ruhig*+2, 450–800 ms post word onset (2050–2400 ms post target onset).**

Starting shortly after 400 ms post word onset, waveforms for MP were more positive-going than those for CP at posterior sites, especially at right-posterior electrodes. Depending on the electrode site, these positivities continued until the end of the segment, or returned closer to baseline levels at varying time points before the end of the segment. There was a main effect of reading (*F*(1,41) = 9.76, *p* < .01.

### 4.2 Time frequency results

**Fig 4:**
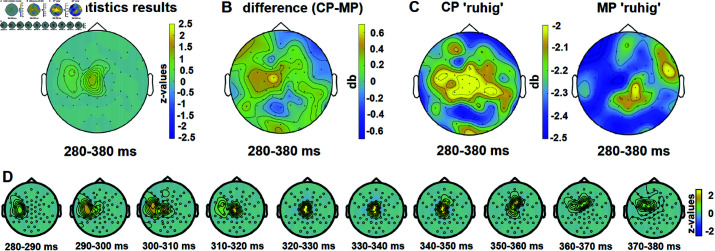
A. The results of Monte Carlo cluster-based permutation tests show significant differences (p = .03, SD = .0054) between CP ruhig and MP ruhig in the low gamma frequency band (30–40 Hz) within the range of 280–380 ms. **B.** Topographies for the CP-MP difference during the time window where significant differences were found (280-380 ms). **C.** Topographies for CP and MP *ruhig* in the low gamma frequency band (30-40 Hz) during the time window of significant differences. **D.** Temporal evolution and scalp distribution of the significant difference between CP and MP *ruhig* conditions.

On the target lexeme, different readings of the word *ruhig* (CP or MP) showed a significant difference (*p* = .03, SD = .0054) in the low gamma frequency range (30—40 Hz) between 280 and 380 ms post-stimulus (Fig 4A). CP *ruhig* showed enhanced power compared to MP *ruhig* (see Fig 4B for the scalp topography representing the CP-MP difference and Fig 4C for the scalp distributions of CP and MP *ruhig* in the low gamma frequency band during the time window, when significant differences were found). Notably, the effect was more pronounced in the left hemisphere from 280 to 320 milliseconds, then shifted to a more centralized pattern from 320 to 360 milliseconds, and finally transitioned to a more lateralized distribution afterwards. A graphical depiction of the results of the time frequency analysis is given in Fig 4.

No significant differences were found on words *ruhig*+1 or *ruhig*+2.

### 4.3 Summary

Results of the ERP and TF analysis show the following pattern:

- A short positive deflection for MP relative to CP readings around 200 ms after word onset. This was visible at posterior sites on *ruhig*, and at anterior sites for *ruhig*+1, with posterior sites displaying the opposite polarity. The difference between readings in this early time window did not persist until word *ruhig*+2.
- More positive-going waveforms for MP than CP readings, especially at posterior/right-posterior sites, beginning around 350 ms on *ruhig*, and around 400 ms on words *ruhig*+1 and *ruhig*+2. The positivities extended for a few hundred milliseconds, with waveforms slowly returning to CP levels, rather than exhibiting a peak-like pattern.
- An enhancement of power in the lower gamma band for CP relative to MP readings, visible in the time window from 280–380 ms post target onset.

## 5 Discussion

**Discussion ERP results.**

Broad positive-going deflections are visible on all three word positions for MP relative to CP readings. These deflections become most clearly visible at (right)-posterior sites. On the target lexeme and the first word following the target lexeme, this was preceded by a short positive peak around 200 ms (with topographies shifting from *ruhig* to *ruhig*+1). We interpret this pattern as a P200–P600 enhancement on *ruhig* and *ruhig*+1, and as a P600 enhancement on *ruhig*+2.

The P200 has been associated with a number of different early language-related processes. Its amplitude is related to the processing of categorical differences for open-class words (most prominently researched for nouns vs. verbs; see [47–51]), most likely due to category-specific morphosyntactic operations (rather than conceptual differences between word classes; [52]). In addition, it has been linked to word frequency (but not predictability), with high-frequency words eliciting smaller P200 amplitudes than low-frequency words ([53], but with stimuli not controlled for word class). For class-ambiguous and meaning-ambiguous words, [54] show that the context affects ERP components related to early lexical stages in word recognition (like the P200), e.g. for biasing an ambiguous word towards a low-frequency reading. In addition to generally reflecting differences in word class and the processing of ambiguity, the P200 amplitude has also been linked to the early detection of pragmatic ambiguity in particular. Examples include the processing of novel literary metaphors [12], politeness violations [13,14] and irony [8,24]; a more detailed outline is following below.

The P600 has been linked to number of unification processes, including syntactic and semantic-thematic integration processes triggered by both ill-formed and well-formed, but cognitively demanding structures (see [55–59] for examples and contrasts to earlier approaches, also [60] for a link of ERP correlates to the MUC framework of sentence processing [61,62]). Since none of these possible factors can be assumed to play a role for our stimuli, we limit our discussion to report of P600 effects linked to the processing of pragmatics and context-dependent expressions. In this literature, the P600 has been linked to the processing of literal vs. non-literal interpretations.

Examples include reports of a P600 enhancement for the processing of metaphors [9,10], indirect requests [5] and irony [6,7,24].

A few studies report P200–P600 enhancement, specifically for increases in pragmatic processing load. Monitoring ERPs elicited to sentence-final words, [24] compared sentences that were literal compared to ironic, disambiguated by a preceding context. For ironic relative to literal conditions, they found an enhanced P200 followed by an enhanced P600 amplitudes. They interpret their findings as showing that non-literal meaning is recognized early if supported by context (reflected in P200 enhancement), and leads to integration of linguistic and contextual information (reflected in P600 enhancement). Despite the difference in stimulus material (ironic/literal vs. MP/CP differences) and point of measurement (sentence-final word vs. a specific target lexeme and its spillover region), the findings of [24] show an interesting parallel to ours, in that they report a P200–P600 pattern for identical stimuli that are only disambiguated by a preceding context, with more positive-going waveforms for the pragmatically more demanding condition. We take this as support for the idea that our findings for *ruhig* do indeed show increased processing load for integrating non-at-issue meaning during ongoing representation building. A P200–P600 enhancement for ironic relative to literal stimuli was also reported by [8]. In this study, ironic and literal interpretations of utterances were triggered by wink emojis or neutral emojis respectively. While the elicited P200 was robust, the size of the P600 response was linked to participants’ likelihood to assume the ironic instead of the literal interpretation. The authors discuss why the P200 does not seem to be affected by the interpretation ultimately adopted by the participants, speculating that it may reflect differences in early visual stimulation by the different emojis. Our own finding of a P200 enhancement elicited by MP relative to CP *ruhig* make this interpretation unlikely, since the target lexeme and the preceding two words are exactly the same. This suggests that the P200 is indeed related to linguistic processes.

Yet another instance of a P200–P600 amplitude increase related to the processing of pragmatics is shown by [14]. Stimuli in their study were sentences with and without politeness violations in Mandarin Chinese. Compared to appropriate sentences, sentences with politeness violations (and without syntactic violations) elicited an enhanced P200 and a centro-parietally distributed positivity lasting from 360 to 866 ms post violation onset. The authors also discuss an earlier study on politeness violations by [13] reporting an enhanced N400; their interpretation of the differences in outcome is that the earlier study use stronger politeness violations that may have triggered additional processes. Our own study did not reveal an influence of the MP-CP contrast on N400 amplitudes, probably reflecting the fact both stimulus conditions are well-formed, reading frequencies are relatively evenly distributed, and the context provides a reliable disambiguation between both readings.

The positivity that we interpret as the P600 become visible relatively early (around 350 ms), however, we are not interpreting it as a P300 enhancement. One reason is that the topography of the effect, especially on the later words, is very much in line with the typical right-central-posterior P600 topography. Another reason is that none of the triggers described in the literature for P300 enhancement (context updating, oddball perception etc.) do not fit our stimuli, which were carefully balanced across conditions and use identical lexical material (see [63] for an overview of the P300 literature). For similar reasons, we are not interpreting the findings for this time window as an increase in N400 amplitude for CP relative to MP; the topography of this effect fits the right-central-posterior P600 topography better than the more central N400 topography. In addition, the only reports of N400 increases for non-literal relative to literal expressions involved strong violations or creative use of language , neither of which are included in our stimuli (see our predictions outlined in the Background section). Instead, we speculate that this positive deflection may reflect semantic-pragmatic integration processes for NAI meaning contributions by MP readings, which become visible in the waveforms at earlier timepoints than those for violation conditions, perhaps due to the fact that they are not overlaid with components reflecting lexical differences or morphosyntactic violations.

The P200 enhancement we see on the target lexeme fits the reports of early detection of pragmatic differences. It would fit the idea that the P200 reflects word class differences, extending the reports in the literature (mainly concerned with contrasts between different open-class words) to the contrast between closed-class modal particles and open class manner adjective/adverb readings of *ruhig*. We could also assume that it reflects the fact that the MP reading of *ruhig* is slightly less frequent than the CP reading, leading to a small P200 enhancement for the former (in line with [53]). All of these explanations suggest that with the support of the preceding disambiguating context, the different readings of *ruhig* are available already at very early stages of word recognition with the support from the preceding context. It also provides an interesting extension to the existing literature on the processing of context-dependent expressions, which often reports ERPs to the target lexeme without including a spillover region into the results.

We interpret the P600 enhancement seen on all three words as reflecting increases in semantic-pragmatic integration cost for MP relative to CP reading. This integration of NAI meaning begins when the MP reading of the target lexeme has correctly been identified, and remains active and costly as the sentence unfolds and integration proceeds. The P600 enhancement for semantic-pragmatic integration is in line with the literature reviewed above. The fact that it remains visible in the spillover region is in line with behavioral findings on comparable stimuli from self-paced readings, which showed strong effects up until word x+3 of the sentence. The early ERP effects of readings are plausible in the context of the wider literature on context-dependent expressions (see discussion above), and add additional evidence gained from highly controlled stimuli. While most of the studies on context-dependent expressions cited focus on ERPs to the target regions, there are some reports of long-lasting effects spanning multiple words (see [5] for indirect requests).

**Discussion time-frequency results.**

As a result of the time-frequency analysis, a significant difference (p = .03) was observed in the low gamma frequency band among different readings of *ruhig*. The central and slightly left-lateralized distribution of the cluster was detected in the time range from 280 to 380 ms, with CP *ruhig* exhibiting more power compared to MP *ruhig*.

In language comprehension, the gamma band is associated with semantic (in contrast to syntactic) unification (see [64,65] for detailed reviews), and has been associated with the processing of incoming bottom-up semantic information [45]. (Some studies associate gamma band activity with the predictability, rather than semantic processing and successful integration of words; see [66], findings in [44,67], and discussions in [65,68]; this discussion is complicated by the fact that a word’s fit with semantic, discourse context and world knowledge all influence its predictability. When considering our own results, it is unlikely that predictability differences play a major role, given that all our stimuli are well-formed, both readings have high and fairly balanced frequencies, and were shown to be a good match in the context in our pretests.)

Low gamma increases have been observed for items with clear and unambiguous referents in contrast to those lacking a clear referent, and have been linked to successful semantic integration (see [69] for pronoun resolution), and for (re)activation of information and semantic integration [40]. We interpret our findings in a similar way, with CP readings of *ruhig* referring to an item with at-issue content (leading to increased synchronization in the gamma band), while MP readings of *ruhig* contribute non-at-issue content (while we would expect this to lead to increased processing demands for semantic unification in the P600 time window, there is less lexical content to be accessed than for CP readings, leading to less synchronization in gamma band activity).

Correlates of semantic processing have been reported both for the low and high gamma band (see, e.g., [70] names that are old vs. new, and coherent vs. incoherent in a given discourse, in a low gamma band from 35-45 Hz). Some accounts suggest that low and high gamma band oscillations reflect different semantic processes. [40] propose that the low gamma band reflects (re)activation of information, while the high gamma band reflects semantic integration; [68] propose that the low gamma band reflects the matching of top-down predictions with bottom-up linguistic input, while the high gamma band reflects the transmission of bottom-up prediction errors to higher processing levels. Since our recording setup does not allow us to monitor high gamma band oscillations, we refrain from an in-depth discussion of potential differences within the gamma range, and limit our discussion to effects associated with low and middle gamma band oscillations.

Interestingly, the literature on irony processing cited above also reports gamma power differences for literal relative to non-literal interpretations for the processing of irony. [7] report increases in theta, alpha and gamma power in different time windows, including an increase in gamma power for ironic vs. literal interpretations from 280–400 ms, beginning left-frontally (280–320 ms) and continueing more centrally (320–370 ms). The authors interpret their findings as reflecting integration during irony processing that become visible well before the P600 amplitude increases, suggesting that the underlying social cognitive processes begin early during language processing. [24] however, do not report gamma power increases for irony processing, showing that the currently available literature does not allow for easy, perhaps simplistic linking between specific power ranges and linguistic phenomena. While our ERP results line up with those of [7] (P200-P600 enhancement for the processing of non-literal/non-at-issue meaning relative to literal/at-issue meaning), the time frequency results don’t: With irony processing, it is the pragmatically more demanding process that shows gamma power enhancement, while in our study, it is the supposedly less demanding at-issue CP condition. A certain amount of discrepancy is not surprising given that both studies investigate very different phenomena; especially, the contrast between MP and CP readings does not involve social pragmatics (like irony does), and interpreting MPs and CPs in context is highly conventionalized.

In light of the literature discussed above, we interpret our time frequency results as reflecting lexical/meaning differences between MP and CP readings, rather than (semantic/pragmatic) integration processes (which we assume to influence the P600).

**Summary and conclusion.**

Our findings show

- Early detection of different readings triggered by the preceding context, reflected in enhanced P200 amplitudes for MP (at this point, both lexical differences and beginning integration differences for at-issue/non-at-issue contributions are plausible - both explanations illustrate how the preceding discourse informs early processing steps).
- Ongoing increases in processing load for MP relative to CP readings, related to integration of non-at-issue information; reflected in enhanced P600 amplitudes for MP, beginning on the target lexeme and still visible on the two following words.
- Lexical/semantic differences between MP (non-at-issue) and CP (at-issue) conditions, reflected in enhanced gamma power for CP conditions.

Our results fit findings from earlier behavioral studies [32,33], showing a long-lasting increase in processing load for NAI relative to AI meaning contributions by MP/CP pairs. This further supports the close link between theoretical description and actual language processing, with the more complex NAI contribution to meaning linked to increased processing workload. The P600 enhancement for MPs relative to CPs suggests that this workload increase is tied to integration/unification related processes. In addition, differences between readings become visible very early in our study, affecting already the P200 amplitude on the target lexeme (while differences between readings only became visible in the spillover region in behavioral studies). At the same time, there is enhanced oscillatory activity in the lower gamma band on the target lexeme for CPs, but not MPs. This lines up with the idea that the counterpart has a clear referent to be retrieved from the lexicon, while the MP reading of *ruhig* does not, its only contribution to sentence meaning being non-at-issue (requiring complex meaning construction reflected in ERPs, but not lexical access to a referent). Our findings thus extend our previous knowledge on the processing of German MPs, providing new insights into its timecourse. Monitoring both ERP and oscillatory activity adds important nuance to our understanding of different types of processing: Whereas earlier studies only revealed the increased processing cost reflecting the integration of NAI meaning provided by MPs, the current study also shows reflections the processing load for AI meaning contributions provided by CPs.

The Standard Pragmatic Model or the Hierarchical Model [16,17] do not lend themselves well to a straightforward discussion of our findings, given that they were formulated to explain the processing of metaphors, rather than highly frequent modal particle - counterpart pairs. Still, if we assume the same underlying mechanisms for literal vs. non-literal and at-issue vs. non-at-issue processing, we would expect NAI meaning to be processed later than AI meanings. Our finding of ERP differences at early latencies on the target lexeme do not fit with this prediction.

In contrast, our findings fit better with predictions of the Direct Access Model [18], which allows a strong influence of the disambiguating context. Under this assumption, the effects found for MP processing could be linked to the construction of complex NAI meanings without first building the less complex AI meaning of the counterpart. In a similar way, the findings fit with the predictions of Graded Salience Model [19,20], which allows for an influence of use frequency in addition to context. This model is particularly attractive when considering the strong influence of use frequencies of different MP-CP pairs in earlier behavioral studies [32]. Future studies should extend our current work by including additional MP-CP pairs with clear reading frequency biases. This will allow further insights into the role of context and its interplay with reading frequencies, both with respect to the neurolinguistic signatures established here and to provide data for more generalized models of context-dependent processing.

Another remaining open issue in the wider literature is how the processing of context-dependent expressions relates to syntactic-semantic processing. For irony, the effects of grammar violations and irony comprehension are additive, suggesting independent processing of both types of information [8]. With politeness markers, one the other hand, the effects of grammar and politeness violations are not additive, suggesting that the processing of grammar has functional primacy over the processing of context-dependent pragmatics (at least the ones tied to a specific morpheme) ([14], see also [21,22] for a formalization). Our current experiment only monitors the processing of contributions to NAI meanings in syntactically and semantically well-formed sentences, and does not allow us to speculate on whether the construction of NAI meanings introduced by MPs functionally depends on syntactic well-formedness. However, our findings lay the groundwork for future studies tackling this very issue.
